# Effect of Konjac Mannan Oligosaccharides on Glucose Homeostasis via the Improvement of Insulin and Leptin Resistance In Vitro and In Vivo

**DOI:** 10.3390/nu11081705

**Published:** 2019-07-24

**Authors:** Di Zhu, Qiaojuan Yan, Yanxiao Li, Jun Liu, Haijie Liu, Zhengqiang Jiang

**Affiliations:** 1 Beijing Advanced Innovation Center for Food Nutrition and Human Health, China Agricultural University, Beijing 100083, China; 2 Bioresource Utilization Laboratory, College of Engineering, China Agricultural University, Beijing 100083, China

**Keywords:** konjac mannan oligosaccharides, insulin resistance, leptin resistance, glucose homeostasis

## Abstract

Functional oligosaccharides, particularly konjac mannan oligosaccharides (KMOS), can regulate glucose metabolism. However, the molecular mechanisms involved in the hypoglycemic effect of KMOS remain largely unknown. Here, the effect of KMOS supplementation on glucose homeostasis was evaluated in both high-fat diet (HFD)-fed C57BL/6J mice and high-glucosamine-induced HepG2 cells. KMOS supplementation remarkably ameliorated the fasting blood glucose, glucose tolerance, and insulin tolerance of HFD-fed mice. Abnormalities of triglyceride and glycogen metabolism in the liver induced by the HFD were reversed by KMOS supplementation. The insulin signaling pathway was activated by KMOS, with stimulation of GLUT2 membrane translocation and glucose uptake in HepG2 cells via the AMPK pathway. Moreover, KMOS suppressed p-mTOR expression and stimulated the GSK-3β/CREB pathway via the AMPK pathway. KMOS significantly upregulated leptin receptor expression and downregulated PTP1B and SOCS3 levels in the liver and brain, with a decreased serum leptin concentration. Phosphorylation of JAK2 and STAT3 in the liver was activated by KMOS supplementation, while the expressions of Sirt1, Tfam, and Pgc1-α in the brain were elevated. Conclusively, KMOS attenuated HFD-induced glucose metabolism dysfunction through the regulation of insulin resistance and leptin resistance. This finding indicates that KMOS have potential value as an anti-hyperglycemic dietary supplement.

## 1. Introduction

Nutrient surplus, eating patterns, reduced weight management, and other unhealthy lifestyles are partially responsible for the rising occurrence of glucose metabolism disorder, resulting in hypertension and cardiovascular diseases [1,2]. Chronic hyperglycemia plays a pivotal role in the progress of metabolic syndromes, notably diabetes and obesity, which imposes a great socioeconomic and medical burden worldwide [3,4]. Hormones, including leptin and insulin, play key roles in maintaining blood glucose homeostasis. Recent studies have revealed that insulin resistance and leptin resistance are characteristic of obesity and type 2 diabetes mellitus (T2DM) [5]. The decreased absorption of glucose from blood to cells in peripheral organs is correlated with the inhibition of insulin action, which leads to insulin resistance [6]. AMP-activated protein kinase (AMPK) regulates lipid metabolism, glucose homeostasis, and inflammation, thus playing a dominant role in targeted therapeutics for chronic metabolic diseases [7]. As a core metabolic pathway of energy regulation, AMPK stimulates glucose uptake in a non-insulin-independent manner and regulates hepatic glycogen synthesis [3]. Leptin manages the central nervous system, glycolipid metabolism, and immune system to affect appetite and energy regulation. Receptors of leptin are widely found in tissues such as the hypothalamus, liver, and fat [8,9]. The Janus kinase (JAK)/signal transducer and activator of transcription (STAT) pathway is a signal transduction pathway of metabolic hormones. Cytokines involved in peripheral organization, such as leptin, IL-6, IL-4, and IFN-γ, are related to the risk of metabolic syndrome [10]. With respect to hepatic metabolic functions, the JAK/STAT pathway can help regulate hepatic glucose production, hepatic steatosis, and insulin resistance [11]. Generally, leptin binding to LepR activates JAK2, which in turn phosphorylates LepR on tyrosine. Subsequently, STAT3 is phosphorylated to allow STAT3 dimerization and translocation to the nucleus [5,12].

For diabetic patients, the treatment of glucagon-like peptide1 receptor agonists is associated with questions surrounding their safety, principally with regard to medullary thyroid cancer, pancreatitis, and pancreatic cancer [13]. Metformin cannot be used in patients with kidney disease because of the risk of lactic acidosis [14]. Furthermore, rosiglitazone is recognized as a cardiovascular-risk-related drug in diabetic patients [15]. In view of the toxic side effects of the common anti-hyperglycemia drugs, the necessity of exploring natural glucose-lowering drugs is imperative. Dietary intervention is regarded as a potential strategy suitable for chronic hyperglycemia treatment via inhibition of inflammation, amelioration of insulin sensitivity, proliferation of healthy intestinal flora, and regulation of glucolipid metabolism homeostasis [2,16]. Many functional foods and dietary supplements have been reported to be effective in the management of metabolic diseases, including plant extracts, fermentation products, and functional oligosaccharides [17]. Functional oligosaccharides are the hydrolytic products of polysaccharides. They cannot be digested in the gastrointestinal tract, but can be utilized as probiotics [2]. As a generally regarded as safe (GRAS) food ingredient, functional oligosaccharides have various beneficial health effects, especially glucose homeostasis regulation [18,19]. Neoagarooligosaccharides prepared by agar hydrolyzation with β-agarase DagA from *Streptomyces coelicolor* regulated adiponectin production and hepatic steatosis, thus exerting anti-obesity and anti-diabetic effects in high-fat diet (HFD)-fed mice [20]. N-acetyl-chitobiose ameliorated the metabolism dysfunction through Erk/p38 MAPK and histone H3 phosphorylation in type 2 diabetic mice [21].

Konjac mannan oligosaccharides (KMOS) are the hydrolytic products of konjac glucomannan, isolated from tubers of *Amorphophallus konjac* K. Koch [22]. They are composed of β-D-mannose and β-D-glucose residues joined together through β-(1→4) glycosidic linkages [23]. KMOS are commonly used in animal feed and as food additives due to their probiotic effects [24]. KMOS supplementation has been reported to help trinitro-benzene-sulfonic acid-induced colitic mice [25], modulate immunity in aged BALB/c mice [26], regulate gut microbiota in diphenoxylate-induced constipated mice, and exert an anti-obesity effect in HFD-fed mice [24,27]. In particular, KMOS improved the function of isolated islets in primary cells of streptozocin-induced diabetic mice [28] and augmented the hypoglycemic effect of metformin (Met) by modulating the gut microbiota [29]. However, the exact molecular mechanisms involved in the hypoglycemic effect of KMOS remain poorly understood.

This study was designed to investigate the potential regulative effects of KMOS on glucose homeostasis in both HFD-fed C57BL/6J mice and high-glucosamine (HG)-induced HepG2 cells. The underlying molecular mechanisms of KMOS against hyperglycemia were elucidated both in vitro and in vivo. Findings from this study will help to expand the application of KMOS as a functional dietary supplement.

## 2. Materials and Methods

### 2.1. Materials and Reagents

KMOS (average molecular weight 342.3-1639.4 Da, mole ratio of mannose to glucose 1.45:1, degree of polymerization 2–10) were supplied by Xi’an Yuansen Biological Technology Co., Ltd. (Xi’an, China). Mannooligosaccharides with a degree of polymerization of 2 to 6 account for ˃80% of KMOS, with 2.1% of mannose [27]. HepG2 cells were purchased from the Type Culture Collection of Chinese Academy of Sciences (Shanghai, China). RPMI 1640 medium, penicillin–streptomycin solution 100× (Vetec reagent grade with 10,000 units penicillin and 10 mg streptomycin/mL), and 2.5% (*w*/*w*) trypsin (2.21 mM EDTA) were obtained from Corning (Manassas, VA, USA). Fetal bovine serum was provided by Biological Industries Inc. (Cromwell, CT, USA). EDTA-free protease inhibitor cocktail tablets and phosphatase inhibitor cocktail tablets were acquired from Roche Diagnostics Ltd. (Mannheim, Germany). Insulin was purchased from Sigma Chemical Co. (St. Louis, MO, USA). Met was obtained from Huazhong Harvey Gene Biological Technology Co., Ltd. (Beijing, China). Compound C was purchased from Selleck (Shanghai, China). ELISA kits for protein tyrosine phosphatase 1B (PTP1B), suppressor of cytokine signaling 3 (SOCS3), brain-derived neurotrophic factor (BDNF), peptide YY (PYY), monocyte chemotactic protein 1 (MCP-1), leptin, and leptin receptor were purchased from Xinle Biotechnology Co., Ltd. (Shanghai, China). The assay kits for glucose, aspartate aminotransferase (AST), alanine aminotransferase (ALT), phosphoenolpyruvate carboxylase kinase (PEPCK), pyruvate kinase (PK), triglyceride (TG), and liver/muscle glycogen were acquired from Nanjing Jiancheng Institute of Biotechnology (Nanjing, China). An insulin ELISA kit was obtained from Abcam (Shanghai, China). A bicinchoninic acid protein assay kit, membrane protein extraction kit, and radio immunoprecipitation assay lysis buffer were purchased from Beyotime Institute of Biotechnology (Shanghai, China). A chemiluminescent electrochemiluminescence assay kit was obtained from Bio-Rad (Hercules, CA, USA).

The primary antibodies against GAPDH, protein kinase B (AKT), p-AKT, Na^+^/K^+^-ATPase α1, p-JAK2, p-STAT3, insulin receptor substrate (IRS)-1, p-IRS-1^Ser307^, AKT substrate of 160 kDa (AS160), p-AS160, p-AMPK, AMPK, p-glycogen synthase kinase-3β (GSK-3β), p-cAMP response element binding protein (p-CREB), mammalian target of rapamycin (mTOR), p-mTOR, and p-p70S6K were purchased from Cell Signaling Technology (Beverly, MA, USA); JAK2, STAT3, and Glucose transporter 2 (GLUT2) were purchased from the Proteintech Group, Inc. (Rosemont, IL, USA); p-IRS-1^Tyr612^ was purchased from Millipore (Billerica, MA, USA). All other chemicals were of analytical grade and were used as received without further purification.

### 2.2. Animal Experimental Design and Sample Collection of Serum and Tissues

A total of 84 two-month-old healthy male C57BL/6J mice were obtained from Vital River Laboratories (Beijing, China). Mice were individually raised in polypropylene cages under standard conditions (12/12 light‒dark cycle, humidity at 55 ± 5%, temperature 25 ± 2 °C). Diets and water were supplied ad libitum. After one adaptive feeding week, mice were randomly assigned to seven groups (*n* = 12/group): (1) control group fed with a standard diet; (2) H-KMOS group fed with a standard diet plus 1200 mg/kg b.w./d KMOS; (3) HFD group fed with HFD (60% kcal from fat); (4) HFD+L-KMOS group fed with HFD plus 400 mg/kg b.w./d KMOS (L-KMOS); (5) HFD+M-KMOS group fed with HFD plus 800 mg/kg b.w./d KMOS (M-KMOS); (6) HFD+H-KMOS group fed with HFD plus 1200 mg/kg b.w./d KMOS (H-KMOS); (7) HFD+Met group fed with HFD plus 400 mg/kg b.w./d Met for 12 weeks. Both KMOS and Met (positive control) were dissolved in drinking water. Compositions of both the standard diet (D12450B) and HFD (D12492) obtained from Keao Xieli Feed Co., Ltd. (Beijing, China) are shown in Table A1. All of the experimental procedures followed Springer’s ethical policies and Chinese national guidelines on the care and use of laboratory animals. The animal handling protocol was approved by the principles of the Institutional Animal Ethics Committee of China Agricultural University (Approval number: 20185001-3).

The body weight, fluid intake, and food intake of the mice were monitored once a week. After fasting for 16 h, all animals were sacrificed. The blood samples were collected by enucleating the eyeball before cervical dislocation and kept at 4 °C for coagulation (4 h). After centrifugation at 1150 *g* and 4 °C for 15 min, the serum was separated out and stored at −80 °C until further analysis. The liver and brain were immediately removed, washed in a phosphate buffer saline solution to remove excess blood, and dried. Tissues were fixed in 10% formalin at 4 °C for morphological observation or stored at −80 °C for subsequent analysis.

### 2.3. Insulin Tolerance Test (ITT) and Oral Glucose Tolerance Test (OGTT)

The ITT and OGTT were carried out at week 10 and week 11, respectively. For ITT, insulin (0.75 U/kg b.w.) was intraperitoneally injected after 6 h fasting. Blood samples were collected and measured at 0, 15, 30, 45, 60, 90, and 120 min after the insulin injection. For OGTT, the overnight-fasted (16 h) mice were orally dosed with D-glucose (2 g/kg b.w., dissolved in water) solution via gavage. Blood samples were collected and measured at 0, 30, 60, 90, and 120 min after the oral dosing with D-glucose. Blood samples were obtained from the tail vein [30], and the blood glucose level was measured using a portable glucometer (ACCU-CHEK^®^ Active, F. Hoffmann-La Roche, Ltd., Switzerland). ITT and OGTT curves were mapped on a graph, and the areas under the curve (AUC) were calculated via the integral method of Origin 2018.

### 2.4. Fasting Blood Glucose and Index of Insulin Resistance

Levels of fasting serum glucose and insulin were detected by the available diagnostic kits or commercial ELISA kits. The degree of insulin resistance was evaluated by the homeostasis model assessment of insulin resistance (HOMA-IR) index, which was calculated using the levels of fasting serum glucose and insulin according to the following formula [31]:

HOMA-IR = fasting insulin (mUI/L) × fasting blood glucose (mmol/L)/22.4.(1)

### 2.5. Serum Biochemical Analysis

Serum levels of AST, ALT, TG, and leptin were analyzed by a Mindray BS-420 Automatic Analyzer (Shenzhen, China) using the available diagnostic kits or commercial ELISA kits based on the manufacturer’s instructions.

### 2.6. Biochemical Analysis of Liver and Brain Tissues

The liver and brain tissues were ground in liquid nitrogen, weighed, and homogenated on ice with 1×PBS (pH 7.4, 10% *w*/*v*). Supernatants of the homogenates were collected for the subsequent analysis after centrifugation at 1150 *g* for 20 min at 4 °C. The contents of PEPCK, PK, TG, and glycogen, the expression levels of PTP1B, SOCS3, and leptin receptor in the liver, as well as the expression levels of leptin receptor, PTP1B, SOCS3, PYY, BDNF, and MCP-1 in the brain were analyzed by a Mindray BS-420 Automatic Analyzer using the available diagnostic kits or commercial ELISA kits following the manufacturer’s instructions.

### 2.7. Histopathology

According to the standardized operation of histopathology, the stored liver tissues were embedded in paraffin after drying, sliced into 5-μm-thick sections, and stained with hematoxylin and eosin (H&E) staining dyes. After dehydration (70%, 90%, and 100% alcohol in turn, 10 s at each level) and clear (xylene for 1 min), the paraffin sections were mounted with Permount and dried at room temperature. An optical microscope at 200× magnification (BM Optical Instrument Manufacturing Co. Ltd., Shanghai, China) was used to image the mounted sections. The representative pictures were taken from five individual liver samples in each group.

### 2.8. Cell Culture and Glucose Uptake

HepG2 cells were maintained in RPMI 1640 medium containing 10% (*v*/*v*) fetal bovine serum and 1% (*v*/*v*) antibiotic–antimycotic solution (100 units/mL penicillin G, 10 g/mL streptomycin, and 25 mg/mL amphotericin B). Cells were cultured in a humidified incubator (MCO-15AC, SANYO Electric CD., Ltd., Osaka, Japan) at 37 °C in an atmosphere of 5% CO_2_. For HG-induced insulin-resistant cell model construction, cells were cultivated with 18 mM glucosamine for 18 h [32]. Prior to experimental treatment, the serum-free medium was used for the starvation of cells for 4 h. Then, cells were cultivated in RPMI 1640 medium containing KMOS (L-KMOS, 0.1 mg/mL; M-KMOS, 0.5 mg/mL; H-KMOS, 1 mg/mL) or Met (1 mg/mL) dissolved in phosphate-buffered saline (the original medium) with or without AMPK inhibitor (Compound C, 20 μmol/L) pretreatment. After 24 h cultivation, the cell culture medium of each experimental group was collected as the final cell supernatant. The glucose contents in both the original medium and the final cell supernatant were measured at 570 nm using a Mindray BS-420 Automatic Analyzer based on the instructions of the glucose diagnostic kit. Glucose uptake was calculated based on the difference between the glucose content of the final cell supernatant and the original medium [32].

### 2.9. Protein Extraction and Western Blotting

After the experimental treatments, HepG2 cells and liver tissues were lysed with a radio immunoprecipitation assay lysis buffer (Midi) (Beyotime Institute of Biotechnology, Shanghai, China) containing a 1/200 dilution of protease and phosphatase inhibitor cocktails for 20 min. Proteins were collected after centrifugation at 21,230 *g* and 4 °C for 10 min. In particular, the membrane protein of HepG2 cells was extracted using the membrane protein extraction kit based on the manufacturer’s instructions. Protein concentration was measured using a bicinchoninic acid protein assay kit.

The collected proteins (50 μg) were separated by sodium dodecyl sulfate/polyacrylamide gel electrophoresis and transferred to 0.45 μm polyvinylidene fluoride membranes (Millipore, Billerica, MA, USA). After blocking with 5% nonfat milk for 2 h, the membranes were incubated with specific primary antibodies (1:1000 dilution) at 4 °C overnight. Membranes were washed three times with tris-buffered saline containing 0.1% Tween 20 and were incubated with the corresponding horse radish peroxidase-conjugated secondary antibodies at room temperature for 2 h. The target protein was detected with a chemiluminescent electrochemiluminescence assay kit and analyzed using the ChemiDoc XRS system (Bio-Rad, Richmond, CA, USA). Quantity One software (Bio-Rad, Richmond, CA, USA) was used to expose and perform gray semi-quantitative analysis.

### 2.10. mRNA Isolation and Gene Expression Quantification

Total RNA was extracted from frozen liver and brain tissues and HepG2 cells using 9767 mRNA extraction kits (Takara, Dalian, Liaoning, China) following the manufacturer’s instructions. Then, 1 µg of total RNA was subjected to the reverse transcription reaction using PrimeScript™ RT Master Mix RR036A (Dalian, Liaoning, China). The cDNA was used as a template to measure the mRNA expression levels using POWER SYBR Green master mix (TaKaARa, Dalian, Liaoning, China). Sirtuin 1 (Sirt1), mitochondrial transcription factor A (Tfam), peroxisome proliferator-activated receptor coactivator 1α (Pgc1-α), glycogen synthase (Gs), Glut2, Gapdh, and β-actin were amplified through the LightCycler^®^ 96 real time-quantitative polymerase chain reaction (RT-qPCR) system (Roche, Mannheim, Germany). The RT-qPCR cycle was as follows: initial hold step, 30 s at 95 °C, followed by 40 cycles of 5 s at 95 °C, 60 °C for 30 s, and 72 °C for 30 s. Melting curves were obtained stepwise from 55 °C to 95 °C. The expression level of each targeted gene was quantified using the comparative method (2^−ΔΔCt^), following normalization with Gapdh (liver and brain tissues) or β-actin (HepG2 cells). The primers for the genes are shown in Table 1. Data were generated from five independent experiments in three replicates for each sample.

### 2.11. Statistics

All data shown are the mean ± standard deviation (SD) for three or more independent experiments. Statistical analyses were performed using one-way analysis of variance (one-way ANOVA) with Tukey’s multiple-range test (SPSS 19.0); *p* < 0.05 was considered statistically significant.

## 3. Results

### 3.1. The Attenuation Effect of KMOS on Body Weight Gain in HFD-Fed C57BL/6J Mice

**Figure 1:**
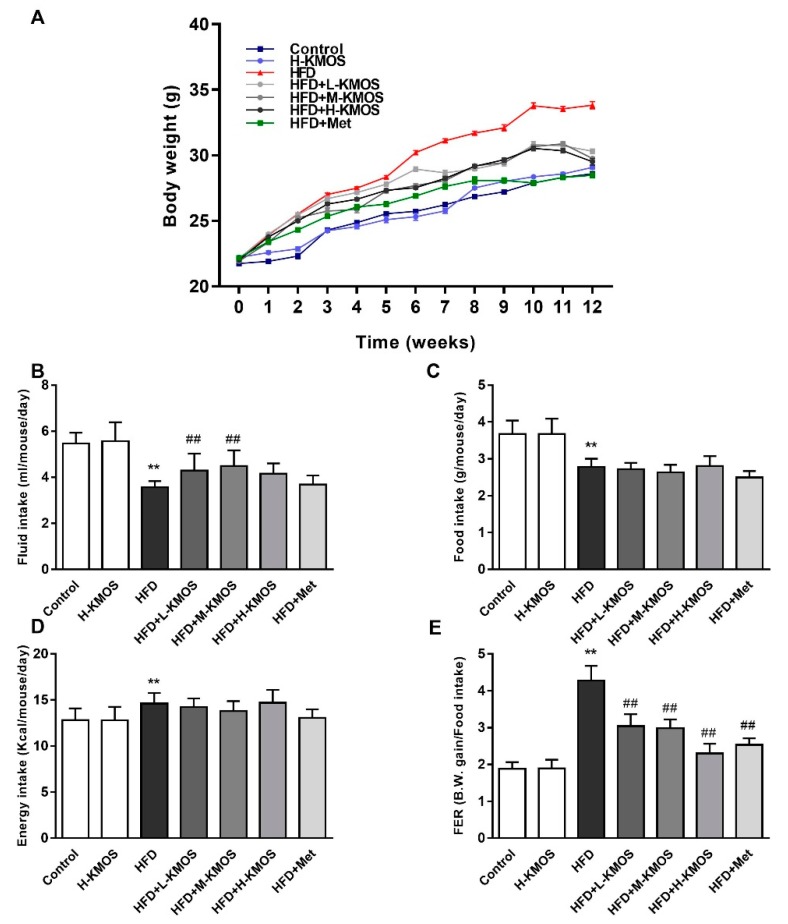
Effect of konjac mannan oligosaccharides (KMOS) supplementation on body weight and other basic properties in HFD-fed C57BL/6J mice. Two-month-old male C57BL/6J mice were randomized into seven groups (*n* = 12 per group): Control, H-KMOS, HFD, HFD+L-KMOS, HFD+M-KMOS, HFD+H-KMOS, and HFD+Met for 12 weeks. Met was used as a positive control. (**A**) Body weight; (**B**) fluid intake; (**C**) food intake; (**D**) energy intake; (**E**) food efficiency ratio (FER). Data are presented as the mean ± standard deviation (SD) (*n* = 12). ** *p* < 0.01 versus the control group, ^##^ *p* < 0.01 versus the HFD group. HFD, high-fat diet (60% kcal from fat); L-KMOS, 400 mg/kg b.w./d KMOS; M-KMOS, 800 mg/kg b.w./d KMOS; H-KMOS, 1200 mg/kg b.w./d KMOS; Met, 400 mg/kg b.w./d metformin.

Compared to the mice in the control group, HFD feeding induced a prominent increase in body weight starting from week 2, and this pattern was seen for the rest of the HFD feeding period (*p* < 0.01, Figure 1A). KMOS (400 mg/kg b.w./d, 800 mg/kg b.w./d, and 1200 mg/kg b.w./d) or Met (400 mg/kg b.w./d, positive control) supplementation significantly lowered body weight from week 6 compared to the mice in the HFD group (*p* < 0.01). The water intake of HFD-fed mice was apparently lower than that in the control group; this pattern was significantly reversed in the L-KMOS+HFD group and M-KMOS+HFD group (*p* < 0.01, Figure 1B). However, there was no marked difference in water intake between the HFD group and the H-KMOS+HFD group (*p* > 0.05). As shown in Figure 1C,D, the food intake and energy intake were not significantly different among all the HFD-fed groups (*p* > 0.05). The food efficiency ratio of mice in the HFD group was higher than that in the control group, and KMOS supplementation significantly decreased the food efficiency ratio in HFD-fed mice (*p* < 0.01, Figure 1E).

### 3.2. Improvement of Glucose Sensitivity and Insulin Sensitivity in HFD-Fed Mice Due to KMOS

**Figure 2:**
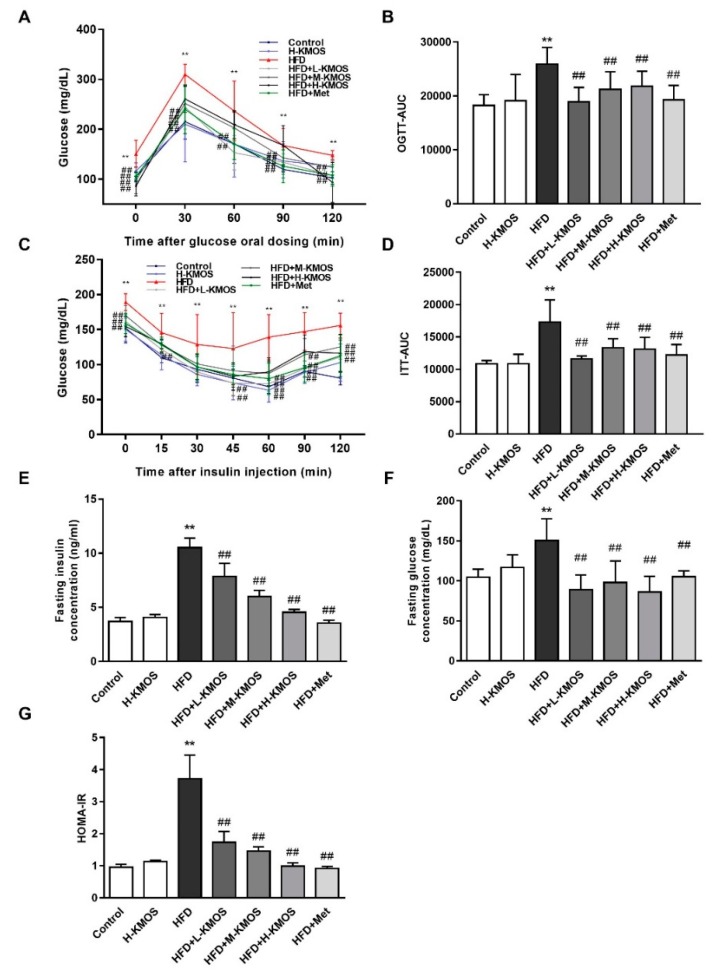
Effect of KMOS supplementation on glucose tolerance and insulin tolerance in HFD-fed mice. (**A**) Oral glucose tolerance test (OGTT); (**B**) areas under the curve (AUC) analysis for OGTT; (**C**) insulin tolerance test (ITT); (**D**) AUC analysis for ITT; (**E**) fasting insulin concentration; (**F**) fasting glucose concentration; (**G**) homeostasis model assessment of insulin resistance (HOMA-IR) index. Data are presented as the mean ± SD (*n* = 5). Values of HOMA-IR index are expressed as the fold changes compared to the control, which was arbitrarily set to 1. ** *p* < 0.01 versus the control group, ^##^ *p* < 0.01 versus the HFD group. HFD, high-fat diet (60% kcal from fat); L-KMOS, 400 mg/kg b.w./d KMOS; M-KMOS, 800 mg/kg b.w./d KMOS; H-KMOS, 1200 mg/kg b.w./d KMOS; Met, 400 mg/kg b.w./d metformin.

As shown by OGTT, HFD-fed mice showed notable high blood glucose after the oral glucose administration (*p* < 0.01). This indicated that glycometabolism disorder was induced by the HFD in mice. KMOS or Met supplementation significantly reduced the glucose excursion and elevated the glucose sensitivity in HFD-fed mice (*p* < 0.01, Figure 2A). The OGTT-AUC value was obviously upregulated by HFD feeding compared to the control group, while OGTT-AUC was significantly decreased by KMOS or Met supplementation (*p* < 0.01, Figure 2B). After insulin injection, the blood glucose levels of the KMOS groups were significantly lower at 60, 90, and 120 min than those of the HFD group (*p* < 0.01). Both KMOS and Met significantly reduced the glucose excursion in HFD-fed mice (*p* < 0.01, Figure 2C). Consistently, statistical significance was observed between the ITT-AUC values of the HFD group and the KMOS groups (*p* < 0.01, Figure 2D).

The HFD caused a notable increase in insulin compared with the control group (*p* < 0.01). L-KMOS, M-KMOS, and H-KMOS significantly downregulated insulin levels in a dose-dependent manner (by 25.4%, 42.9%, and 56.9%, respectively) compared with the HFD group (*p* < 0.01, Figure 2E). Furthermore, the HFD induced a prominent increase in fasting blood glucose level compared to the control group (*p* < 0.01). L-KMOS, M-KMOS, and H-KMOS supplementation significantly decreased the fasting blood glucose level (by 40.7%, 34.9%, and 42.8%, respectively) (*p* < 0.01, Figure 2F). The HFD significantly elevated the HOMA-IR index about 2.9-fold compared with the control group (*p* < 0.01). In contrast with the HFD group, the HOMA-IR index was significantly attenuated in a dose-dependent manner (by 53.3%, 61.0%, and 73.3%, respectively) in the L-KMOS, M-KMOS, and H-KMOS+HFD groups (*p* < 0.01, Figure 2G).

### 3.3. Alleviation of Liver Damage and Hepatic Glucose Metabolism Disorder by KMOS

**Figure 3:**
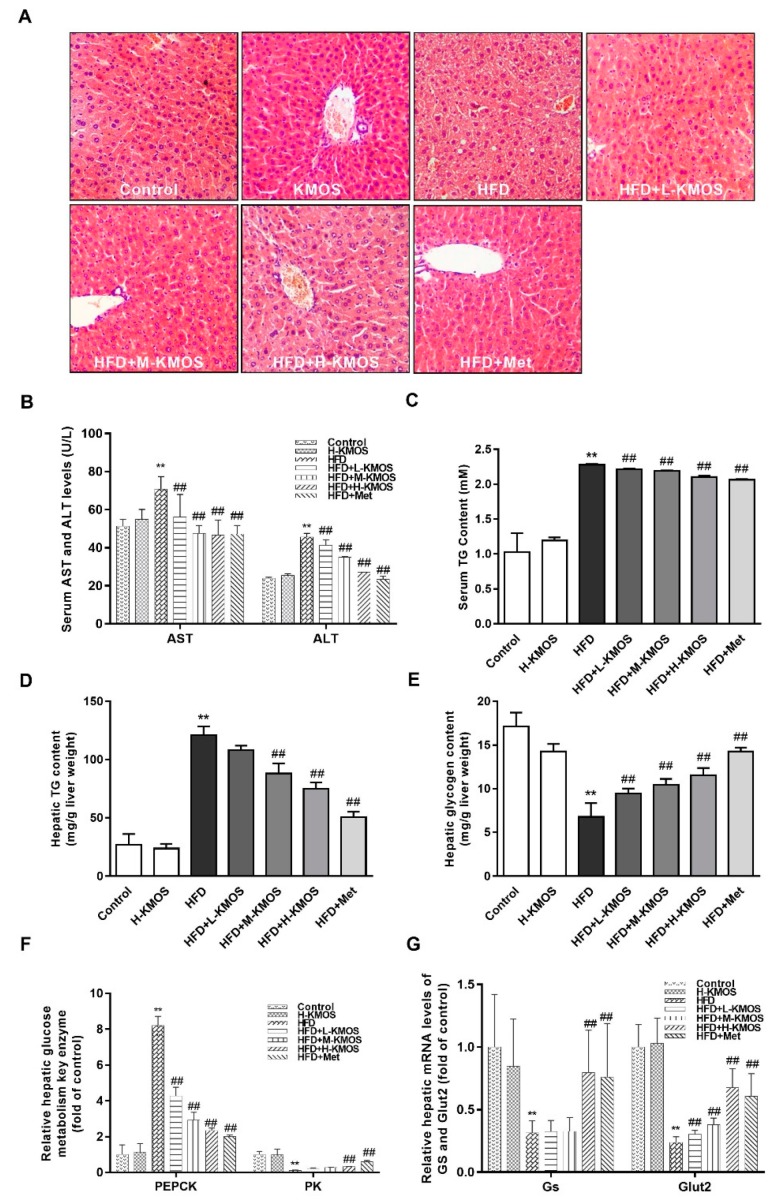
Effect of KMOS on liver damage and hepatic glucose metabolism disorder. (**A**) Representative photomicrographs of hematoxylin-eosin (H&E) staining in the liver (magnification 200×); (**B**) the liver dysfunction indices of activities of aspartate aminotransferase (AST) and alanine aminotransferase (ALT); (**C**) serum triglyceride (TG) content; (**D**) hepatic TG content; (**E**) hepatic glycogen content; (**F**) the vitality of hepatic glycolytic and gluconeogenesis key enzymes of phosphoenolpyruvate carboxylase kinase (PEPCK) and pyruvate kinase (PK) in the liver; (**G**) mRNA levels of glycogen synthase (Gs) and glucose transporter 2 (Glut2) in the liver. Values are expressed as the fold change compared to the control, which was arbitrarily set to 1. Data are presented as the mean ± SD (*n* = 5). ** *p* < 0.01 versus the control group, ^##^ *p* < 0.01 versus the HFD group. HFD, high-fat diet (60% kcal from fat); L-KMOS, 400 mg/kg b.w./d KMOS; M-KMOS, 800 mg/kg b.w./d KMOS; H-KMOS, 1200 mg/kg b.w./d KMOS; Met, 400 mg/kg b.w./d metformin.

H&E staining showed that the HFD-induced changes in cell morphology included swollen, disordered, and increased intracellular vacuoles (Figure 3A). KMOS or Met supplementation depleted HFD-induced hepatic pathological changes. In the HFD group, serum ALT and AST activities were significantly increased compared to the control group (*p* < 0.01). However, co-administration of KMOS with HFD significantly lowered the serum ALT and AST activities in a dose-dependent manner, as compared with the HFD group (*p* < 0.01, Figure 3B). Lipid accumulation was induced by the HFD in the liver, leading to liver damage. The serum TG content was significantly downregulated by KMOS in a dose-dependent manner in HFD-fed mice (*p* < 0.01, Figure 3C). M-KMOS or H-KMOS supplementation also inhibited HFD-induced hepatic fat accumulation with a significantly decreased hepatic TG content (*p* < 0.01, Figure 3D).

The reduction of glycogen storage, which contributed to glycemic control, was significantly enhanced by L-KMOS, M-KMOS, and H-KMOS supplementation in a dose-dependent manner (by 39.8%, 54.6%, and 70.9%, respectively) in HFD-fed mice (*p* < 0.01, Figure 3E). To investigate the function of liver on blood glucose control, key enzymes in the liver homogenate for glycolysis and gluconeogenesis were analyzed. Compared with the control group, the HFD significantly elevated the PEPCK level and decreased the PK level (*p* < 0.01). H-KMOS supplementation significantly reduced PEPCK and activated PK compared to the HFD group (*p* < 0.01, Figure 3F). H-KMOS supplementation significantly alleviated the decrease of Gs mRNA expression induced by the HFD (*p* < 0.01, Figure 3G). This indicated that glycogen synthesis was reversed by KMOS in HFD-fed mice.

Moreover, the mRNA expression of Glut2, which reflected the degree of hepatic insulin sensitivity, was obviously suppressed in HFD-fed mice compared to the control group. KMOS supplementation significantly enhanced the Glut2 mRNA expression in a dose-dependent manner in the livers of HFD-fed mice (*p* < 0.01, Figure 3G).

### 3.4. Regulation Effect of KMOS on Glucose Metabolism via the AMPK Pathway and Insulin Signaling Pathway

**Figure 4:**
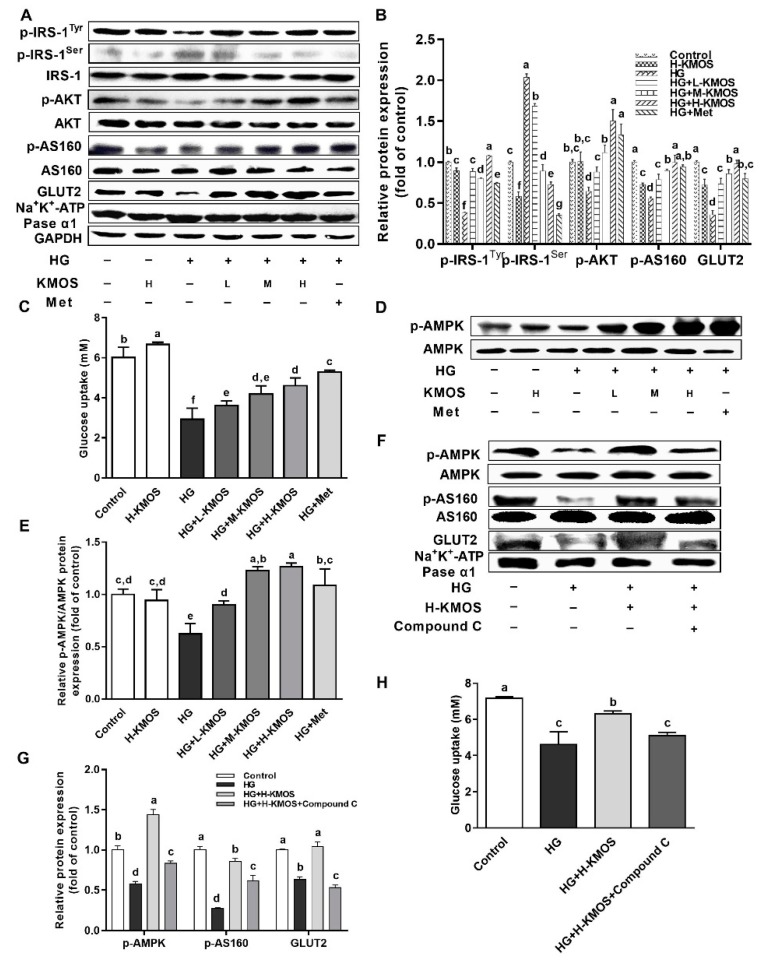
Effect of KMOS on glucose metabolism via the insulin signaling pathway and AMP-activated protein kinase (AMPK) pathway. The incubation of HepG2 cells with 18 mM glucosamine for 18 h was used for the construction of the high-glucosamine (HG)-induced insulin-resistant cell model. After starvation for 2 h with serum-free medium, cells were treated with different concentrations of KMOS (L-KMOS, 0.1 mg/mL; M-KMOS, 0.5 mg/mL; H-KMOS, 1 mg/mL) or Met (1 mg/mL metformin) for 24 h. (**A**) Protein expression in the insulin signaling pathway, including insulin receptor substrate (IRS-1), p-IRS-1^Tyr612^, p-IRS-1^Ser307^, protein kinase B (AKT), p-AKT, AKT substrate of 160 kDa (AS160), p-AS160, membrane GLUT2, and membrane NA^+^/K^+^-Pase α1; (**B**) densitometry quantification of the expressed protein bands of the insulin signaling pathway; (**C**) glucose uptake; (**D**) protein expressions of AMPK and p-AMPK; (**E**) densitometry quantification of the expressed protein bands of p-AMPK and AMPK; (**F**) protein expressions of p-AMPK, AMPK, p-AS160, AS160, membrane GLUT2, and membrane NA^+^/K^+^-Pase α1 in the presence of AMPK inhibitor Compound C (20 μmol/L); (**G**) densitometry quantification of the expressed protein bands in the presence of AMPK inhibitor Compound C; (**H**) glucose uptake in the presence of AMPK inhibitor Compound C. Met was used as a positive control. Results are expressed as the mean ± SD of at least three replicates. Values with different superscripts are significantly different at *p* < 0.05.

The regulative effect of KMOS on insulin resistance was investigated using an HG-induced insulin-resistant HepG2 cell model. A high concentration of glucosamine obviously prevented the action of insulin and blocked the insulin signaling pathway. The phosphorylation of IRS-1^Tyr612^, AKT, and AS160 was significantly suppressed by a high concentration of glucosamine, resulting in remarkably limited glucose uptake and insulin resistance in HepG2 cells (*p* < 0.01). Compared with the HG group, KMOS supplementation significantly increased the phosphorylation of IRS-1^Tyr612^, AKT, and AS160, while the phosphorylation of IRS-1^Ser307^ was significantly reduced (*p* < 0.01). KMOS significantly reversed HG-induced reduction of GLUT2 expression on the plasma membrane in a dose-dependent manner (*p* < 0.01, Figure 4A,B). A high concentration of glucosamine dramatically reduced glucose uptake by 51.1% compared with the untreated cells (*p* < 0.01). L-KMOS, M-KMOS, and H-KMOS significantly upregulated glucose uptake in a dose-dependent manner (by 23.1%, 42.6%, and 56.9%, respectively) compared to the HG group (*p* < 0.01, Figure 4C). Compared with the untreated cells, the AMPK pathway, a non-insulin-mediated regulatory pathway for glucose uptake management, was significantly suppressed in HG-treated cells (*p* < 0.01). Compared with the HG group, KMOS significantly attenuated the HG-induced reduction of AMPK phosphorylation in a dose-dependent manner (*p* < 0.01, Figure 4D,E). Compared with the H-KMOS+HG group, pretreatment with Compound C (AMPK inhibitor) significantly suppressed the phosphorylation of AMPK and AS160 and membrane translocation of GLUT2 (*p* < 0.01, Figure 4F,G). Consistently, after treatment with Compound C and H-KMOS, glucose uptake was significantly decreased by 19.2% compared with HG-induced insulin-resistant cells treated with H-KMOS only (*p* < 0.01, Figure 4H).

### 3.5. Management of KMOS with Respect to mTOR and the Glycogen Metabolic Pathway via the AMPK Pathway

**Figure 5:**
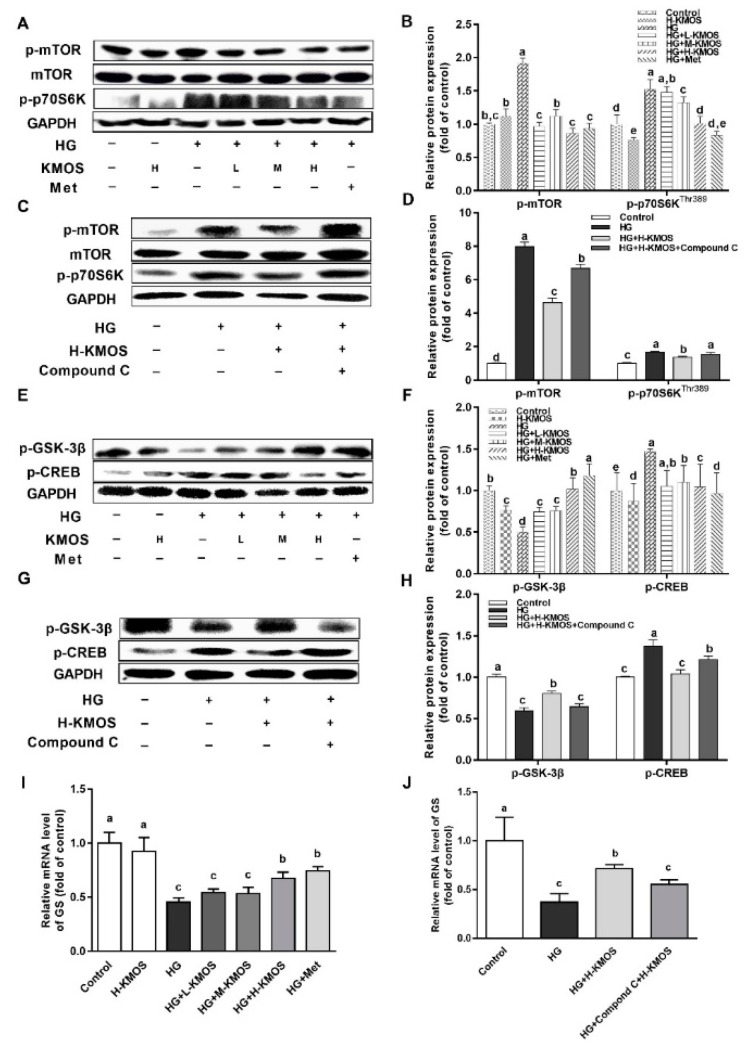
Regulative effect of KMOS on the mammalian target of rapamycin (mTOR) pathway and the glycogen metabolic pathway via the AMP-activated protein kinase (AMPK) pathway. (**A**) Protein expressions of mTOR, p-mTOR, and p-p70S6K in HepG2 cells; (**B**) densitometry quantification of the expressed protein bands of p-mTOR, mTOR, and p-p70S6K; (**C**) protein expressions of p-mTOR, mTOR, and p-p70S6K in the presence of the AMPK inhibitor of Compound C (20 μmol/L); (**D**) densitometry quantification of the expressed protein bands of p-mTOR, mTOR, and p-p70S6K in the presence of the AMPK inhibitor of Compound C; (**E**) protein expressions of p-glycogen synthase kinase-3β (GSK-3β) and p-cAMP response element binding protein (CREB) in HepG2 cells; (**F**) densitometry quantification of the expressed protein bands of p-GSK-3β and p-CREB; (**G**) protein expressions of p-GSK-3β and p-CREB in the presence of the AMPK inhibitor Compound C; (**H**) densitometry quantification of the expressed protein bands of p-GSK-3β and p-CREB in the presence of the AMPK inhibitor Compound C; (**I**) mRNA levels of glycogen synthase (Gs) in the HepG2 cells; (**J**) mRNA levels of Gs in the presence of the AMPK inhibitor of Compound C. Values are expressed as the fold changes compared to the control, which was arbitrarily set to 1. Results are expressed as the mean ± SD of at least three replicates. Values with different superscripts are significantly different at *p* < 0.05. HG, high-glucosamine (18 mM glucosamine); L-KMOS, 0.1 mg/mL KMOS; M-KMOS, 0.5 mg/mL KMOS; H-KMOS, 1 mg/mL KMOS; Met, 1 mg/mL metformin.

HG significantly activated the phosphorylation of mTOR and p70S6K (the downstream substrate of mTOR) in HepG2 cells (*p* < 0.01). KMOS significantly downregulated the phosphorylation of mTOR and p70S6K compared to the HG group (*p* < 0.01, Figure 5A,B). However, H-KMOS plus Compound C significantly increased the phosphorylation of mTOR and p70S6K compared with the HG-induced insulin-resistant cells treated with H-KMOS only (*p* < 0.01, Figure 5C,D). When treated with HG, GSK-3β phosphorylation was significantly suppressed, while CREB phosphorylation was significantly activated in HepG2 cells compared to the untreated cells (*p* < 0.01). KMOS supplementation significantly downregulated CREB phosphorylation and upregulated GSK-3β phosphorylation (*p* < 0.01, Figure 5E,F). H-KMOS plus Compound C significantly attenuated GSK-3β phosphorylation, while CREB phosphorylation was significantly elevated compared with the HG-induced insulin-resistant cells treated with H-KMOS only (*p* < 0.01, Figure 5G,H). Subsequently, the mRNA expression of Gs was significantly activated by KMOS in HG-induced insulin-resistant cells (*p* < 0.01, Figure 5I). Similarly, H-KMOS plus Compound C significantly downregulated Gs mRNA expression compared with the HG-induced insulin-resistant cells treated with H-KMOS only (*p* < 0.01, Figure 5J).

### 3.6. Alleviation of Hepatic Leptin Resistance by KMOS

**Figure 6:**
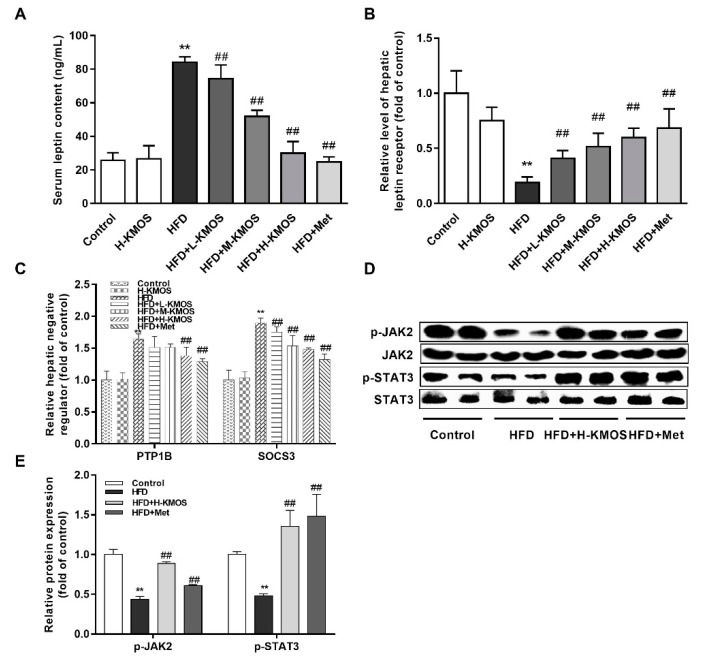
Effect of KMOS on hepatic leptin resistance. (**A**) Serum leptin content; (**B**) expression of leptin receptor in the liver; (**C**) levels of protein tyrosine phosphatase 1B (PTP1B) and suppressor of cytokine signaling 3 (SOCS3) expression in the liver; (**D**) expressions of Janus kinase 2 (JAK2), p-JAK2, signal transducer and activator of transcription 3 (STAT3), and p-STAT3 in the liver; (**E**) densitometry quantification of the expressed protein bands of p-JAK2, JAK2, p-STAT3, and STAT3. Values are expressed as the fold changes compared to the control, which was arbitrarily set to 1. Data are presented as the mean ± SD (*n* = 5). ** *p* < 0.01 versus the control group, ^##^ *p* < 0.01 versus the HFD group. HFD, high-fat diet (60% kcal from fat); L-KMOS, 400 mg/kg b.w./d KMOS; M-KMOS, 800 mg/kg b.w./d KMOS; H-KMOS, 1200 mg/kg b.w./d KMOS; Met, 400 mg/kg b.w./d metformin.

The serum leptin level was significantly upregulated in the HFD group compared to the control group (*p* < 0.01). Compared to the HFD group, L-KMOS, M-KMOS, and H-KMOS significantly downregulated the HFD-induced elevation of the leptin level (by 11.7%, 38.4%, and 64.4%, respectively) in a dose-dependent manner (*p* < 0.01, Figure 6A), indicating the alleviation of leptin resistance by KMOS. Co-administration of KMOS with the HFD significantly activated the expression of the leptin receptor in the liver in a dose-dependent manner compared to the HFD group (*p* < 0.01, Figure 6B). The expression of leptin metabolism-related factors SOCS3 and PTP1B was significantly upregulated by the HFD compared with the control group (*p* < 0.01). In combination with the HFD, H-KMOS significantly reversed the increased expression of SOCS3 and PTP1B in HFD-fed mice (*p* < 0.01, Figure 6C). The HFD significantly suppressed the JAK/STAT pathway, while the phosphorylation of JAK2 and STAT3 was significantly activated after co-administration with H-KMOS supplementation (*p* < 0.01, Figure 6D,E).

### 3.7. Protective Effect of KMOS against Leptin Resistance and Abnormal Metabolism in the Brain

**Figure 7:**
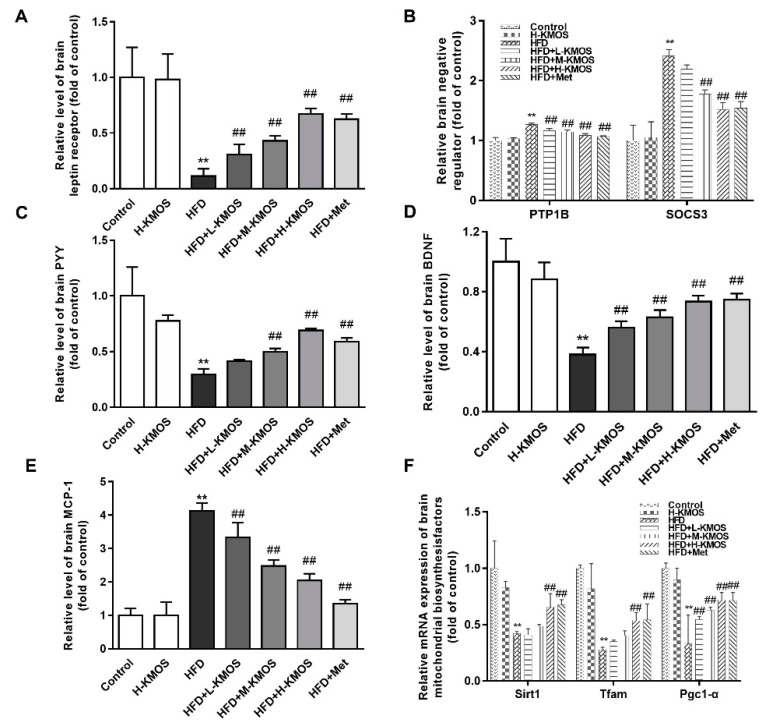
Effect of KMOS on leptin resistance and abnormal metabolism in the brain. (**A**) Leptin receptor expression; (**B**) expression of protein tyrosine phosphatase 1B (PTP1B) and suppressor of cytokine signaling 3 (SOCS3); (**C**) peptide YY (PYY) content; (**D**) brain-derived neurotrophic factor (BDNF) expression level; (**E**) monocyte chemotactic protein 1 (MCP-1) expression level; (**F**) mRNA levels of mitochondrial metabolism-related genes of sirtuin 1 (Sirt1), mitochondrial transcription factor A (Tfam), and peroxisome proliferator-activated receptor coactivator 1-α (Pgc1-α). Data are presented as the mean ± SD (*n* = 5). Values are expressed as the fold changes compared to the control, which was arbitrarily set to 1. ** *p* < 0.01 versus the control group, ^##^ *p* < 0.01 versus the HFD group. HFD, high-fat diet (60% kcal from fat); L-KMOS, 400 mg/kg b.w./d KMOS; M-KMOS, 800 mg/kg b.w./d KMOS; H-KMOS, 1200 mg/kg b.w./d KMOS; Met, 400 mg/kg b.w./d metformin.

Compared to the HFD group, co-administration of KMOS and the HFD significantly reversed the HFD-induced decrease of leptin receptor expression in a dose-dependent manner in the brain (*p* < 0.01, Figure 7A). Expression of leptin metabolism-related negative regulatory factors of PTP1B and SOCS3 was significantly reversed by KMOS supplementation compared to the HFD group (*p* < 0.01, Figure 7B). PYY, an appetite hormone, was significantly enhanced in the M-KMOS or H-KMOS group compared to the HFD group (*p* < 0.01, Figure 7C). Co-administration of KMOS with the HFD dramatically attenuated the HFD-induced suppression of BDNF expression in a dose-dependent manner (*p* < 0.01, Figure 7D). For HFD-induced inflammatory responses, co-administration of KMOS significantly inhibited the MCP-1 level in a dose-dependent manner (Figure 7E). The HFD inhibited the expression of key genes involved in the brain mitochondrial metabolism. However, the gene expression of Sirt1, Tfam, and Pgc1-α was significantly activated by H-KMOS supplementation in HFD-fed mice (*p* < 0.01, Figure 7F).

## 4. Discussion

HFD-fed mice with glucolipid metabolism disorder will develop obesity, hyperglycemia, hyperinsulinemia, and T2DM, and they are broadly used for preliminary functional validation studies of bioactive compounds [5,8,33]. To explore the functional potential of KMOS administration as a lifestyle approach for the regulation of glucose metabolism, we explored the anti-hyperglycemic effects of KMOS in both HFD-fed mice and HG-induced insulin-resistant HepG2 cells, as well as the molecular mechanisms involved.

Through the co-administration of KMOS with the HFD in two-month-old healthy C57BL/6J mice, we investigated the preventive efficacy of KMOS supplementation in glucose metabolic disorder mitigation. Administration of KMOS significantly reduced the body weight (Figure 1) and blood glucose level of HFD-fed mice (Figure 2). HFD-induced impairment of insulin and glucose tolerance was also attenuated by KMOS supplementation, as indicated by ITT and OGTT, respectively. Metabolic stressors, including energy-dense and high-fat diets, are reportedly associated with the promotion of obesity, insulin resistance, and diabetes in humans [34]. Abnormal body weight, fasting glucose, OGTT, HOMA-IR index, and hepatic and serum TG contents are perceived as essential phenomena to evaluate the progression of obesity and T2DM in human patients [35,36,37]. In this study, these key indicators were evaluated in the HFD-fed mice with or without KMOS supplementation. These findings, coupled with existing researches, demonstrated the potential for the utilization of KMOS as a dietary supplement to maintain glucose homeostasis.

In glycemic control, the liver plays a significant role between meals through glucose transformation regulation [31]. Glucose fluxes from gluconeogenesis, glycogen synthesis by liver and muscle, hepatic glycogenolysis, glycolysis, and other pathways together contribute to the net hepatic glucose production [38]. In T2DM patients, the serum ALT level was restored to normal after the consumption of 100 mg/day fructo-oligosaccharides for eight weeks [39]. KMOS also reversed the liver dysfunction (AST and ALT, potential predictors of human T2DM risk [40]) induced by the HFD, as confirmed by histopathology staining (Figure 3A,B). In this process, the lipid aggregation induced by the HFD might contribute to the development of hepatic steatosis and liver damage (Figure 3C,D). However, KMOS has the potential to inhibit hepatic lipid accumulation and to attenuate liver steatosis. As obesity remains a key contributor to T2DM, alternative preventive therapies that target hepatic steatosis and lipid-induced hepatic insulin resistance are necessary for the reduction of hyperglycemia risks. Liver damage is associated with abnormal metabolism and thus contributes to glucose metabolic disorders [38]. KMOS regulated the gene expression of Gs, the key rate-limiting enzyme of gluconeogenesis and glycolytic, and subsequently affected glycogen accumulation (Figure 3E‒G). KMOS have been reported to exhibit indirect glycemic regulation through the proliferation of intestinal probiotics [29]. Interestingly, Glut2 mRNA expression in the liver was regulated after co-administration of KMOS, resulting in attenuated insulin resistance (Figure 3G). Therefore, we speculated that KMOS can directly regulate blood glucose through the regulation of hepatic glucose metabolism.

Insulin resistance is a key risk factor for the development of obesity and T2DM. For a given dose of insulin, the peripheral organization or cells cannot respond appropriately, which is known as insulin resistance [41]. Activation of IRS subsequently triggers the increase of PI3K/AKT and the insulin/IGF-1 signaling pathway (IIS), leading to GLUT membrane translocation. The phosphorylation of AS160 promotes basal GLUT exocytosis and inhibits gluconeogenesis in the liver [42]. Insulin resistance was exhibited in both the muscles and livers of AS160-knockout mice [43]. In HG-induced HepG2 cells, KMOS were found to activate the IIS, stimulate membrane translocation of GLUT2, and subsequently improve glucose uptake capacity (Figure 4A–C). In addition to IIS, AMPK, a control switch of cellular energy status, also impacts lipid metabolism, glucose homeostasis, and inflammation [7]. Fructo-oligosaccharides were reported to activate the AMPK pathway to maintain glucose homeostasis and alter gut microbiota composition as well as the short-chain fatty acids content [2]. Moreover, AMPK is localized to glycogen of more than five glucose units in intact cells and in vitro by β1-CBM, a carbohydrate-binding module [44,45]. This indicates that functional oligosaccharides can directly regulate glucose metabolism via the activation of the AMPK pathway in the liver. KMOS significantly activated the phosphorylation of AMPK (Figure 4D,E). AS160 is the substrate not only for AKT but also for AMPK in the regulation of GLUT membrane translocation [42,46,47]. Treatment in combination with an AMPK inhibitor (Compound C) resulted in decreased phosphorylation of AS160, membrane translocation of GLUT2, and glucose uptake compared to treatment with KMOS only (Figure 4F–H). As a downstream effector of the AMPK pathway, mTOR can suppress hepatic gluconeogenesis and lipogenesis. Moreover, mTOR negatively impacts its upstream IRS pathway through direct phosphorylation of specific residues [48]. Here, the mTOR-S6K1 pathway was remarkably suppressed by KMOS via the AMPK pathway (Figure 5A–D). GSK-3β is regarded as the downstream effector of the mTOR pathway [49]. In HepG2 cells, glycogen storage and glucose production are generally considered to be regulated through AMPK-mediated GSK3β and CREB phosphorylation [50]. Consistently, KMOS reversed the phosphorylation of GSK-3β and CREB, as well as Gs mRNA expression, via the AMPK pathway in HG-induced HepG2 cells. Thus, KMOS regulated hepatic insulin resistance and glycometabolism via the AMPK pathway and its downstream pathways of mTOR and GSK-3β/CREB (Figure 5E‒J).

Leptin is another key hormone associated with blood glucose level and appetite regulation [51]. Obese patients are considered leptin resistant with abnormal leptin receptor expression in the central hypothalamus and peripheral tissues [9]. Peripheral metabolic abnormalities were improved by liver-specific overexpression of exogenous leptin receptor-b in leptin receptor-b-deficient fa/fa rats [52]. The JAK/STAT pathway is implicated in obesity and chronic metabolic diseases, serving as an important adjuster for growth factor hormones and cytokines of leptin, IL-6, and IFN-γ [11]. Chitosan oligosaccharide capsules activated the hepatic leptin receptor-b and leptin signal pathway (JAK2/STAT3 pathway) in HFD-fed rats [53]. Similarly, KMOS supplementation significantly downregulated serum leptin levels and upregulated leptin receptor expression in the liver and brain (Figure 6A,B and Figure 7A). Additionally, the JAK2/STAT3 pathway was effectively activated by KMOS in the livers of HFD-fed mice (Figure 6D,E). In the leptin- and insulin-related pathways, PTP1B and SOCS3 are negative regulators and make major contributions to leptin and insulin resistance [54,55]. The HFD induced the increased expression of PTP1B and SOCS3 in the liver and brain was suppressed by KMOS supplementation (Figure 6C and Figure 7B). Moreover, KMOS regulated leptin and PYY levels in the brain (Figure 7C), which function together as the central regulators of appetite and energy homeostasis [56]. HFD feeding elicited injury in the mouse hippocampus [31], which is considered to be associated with learning impairment and memory decline [57]. KMOS attenuated the HFD-induced suppression of the expression of BDNF (Figure 7D). Hormone metabolic disorders in the brain can be associated with oxidative stress and abnormal mitochondrial metabolism induced by HFD [58]. The MCP-1 level and the mRNA levels of the brain mitochondrial biosynthesis factors of Sirt1, Tfam, and Pgc1-α were notably reversed by KMOS in HFD-fed mice (Figure 7E,F). Therefore, KMOS regulated HFD-induced leptin resistance and leptin-related metabolism in both the liver and brain.

## 5. Conclusions

KMOS supplementation improved glucose metabolism in HFD-fed C57BL/6J mice with decreased fasting blood glucose and HOMA-IR index, thus ameliorating abnormal hepatic glucose metabolism. Insulin resistance was regulated by KMOS via the AMPK pathway, the insulin signaling pathway, and the downstream pathways of the mTOR and glycogen metabolic pathways in HG-induced insulin-resistant HepG2 cells. KMOS reversed leptin resistance by the activation of leptin receptor and the suppression of leptin regulatory inhibitors in both the liver and brain. Additionally, the stimulation of the JAK2/STAT3 pathway in the liver and the elevation of mitochondrial biosynthesis in the brain also contributed to the management of leptin metabolism by KMOS. Therefore, KMOS could potentially be utilized as an alternative dietary intervention for the delay of obesity, diabetes, and their complications.

## Figures and Tables

**Table 1:** Primer sequences used for semi-quantitative RT-qPCR analysis.

	Forward Primer	Reverse Primer
Mouse-Sirt1	TCTGTCTCCTGTGGGATTCC	GATGCTGTTGCAAAGGAACC
Mouse-Tfam	GCTTCCAGGGGGCTAAGGAT	CCCAATCCCAATGACAACTC
Mouse-Pgc1-α	GAAAGGGCCAAACAGAGAGA	GTAAATCACACGGCGCTCTT
Mouse-Gs	CCATGAACAGCAAGGGTTGTAA	TGGAAGTGGGCAACCACATA
Mouse-Glut2	TCAGAAGACAAGATCACCGGA	GCTGGTGTGACTGTAAGTGGG
Mouse-Gapdh	TGGAGAAACCTGCCAAGTATGA	TGGAGAAACCTGCCAAGTATGA
Human-Gs	GCCTTTCCAGAGCACTTCAC	CTCCTCGTCCTCATCGTAGC
Human-β-actin	TGGATCAGCAAGCAGGAGTA	TCGGCCACATTGTGAACTTT

Sirt 1, sirtuin 1; Tfam, mitochondrial transcription factor A; Pgc1-α, peroxisome proliferator-activated receptor coactivator 1-α; Gs, glycogen synthase; Glut2, glucose transporter 2.

## Abbreviations

AKT, protein kinase B; ALT, alanine aminotransferase; AMPK, AMP-activated protein kinase; AS160, AKT substrate of 160 kDa; AST, aspartate aminotransferase; AUC, areas under curve; BDNF, neurotrophic factor; CREB, cAMP response element binding protein; GLUT2, glucose transporter 2; Gs, glycogen synthase; GSK-3β, glycogen synthase kinase-3β; H&E, hematoxylin-eosin; HFD, high-fat diet; HG, high-glucosamine; HOMA-IR, homeostasis model assessment of insulin resistance; IIS, insulin/IGF-1 signaling pathway; IRS, insulin receptor substrate; ITT, insulin tolerance test; JAK/STAT, Janus kinase/signal transducer and activator of transcription; KMOS, konjac mannan oligosaccharides; MCP-1, monocyte chemotactic protein 1; Met, metformin; mTOR, mammalian target of rapamycin; OGTT, oral glucose tolerance test; PEPCK, phosphoenolpyruvate carboxylase kinase; PK, pyruvate kinase; PTP1B, protein tyrosine phosphatase 1B; PYY, peptide YY; RT-qPCR, real time-quantitative polymerase chain reaction; Sirt1, sirtuin 1; SOCS3, suppressor of cytokine signaling 3; T2DM, type 2 diabetes mellitus; Tfam, mitochondrial transcription factor A; TG, triglyceride.

## Appendix A

**Table A1:** Composition of experimental diets.

Ingredient	D12450B (Control)		D12492 (HFD)	
	g	kcal	g	kcal
Casein, 80 Mesh	200	800	200	800
L-Cystine	3	12	3	12
Corn Starch	315	1260	0	0
Maltodextrin 10	35	1260	125	500
Sucrose	350	1400	68.8	275.2
Cellulose, BW200	50	0	50	0
Soybean Oil	25	225	25	225
Lard	20	180	245	2205
Mineral Mix, S10026	10	0	10	0
Dicalcium Phosphate	13	0	13	0
Calcium Carbonate	5.5	0	5.5	0
Potassium Citrate, 1 H_2_O	16.5	0	16.5	0
Vitamin Mix, V10001	10	40	10	40
Choline Bitartrate	2	0	2	0
FD&C Blue Dye #1	0.05	0	0.05	0
Total (g) and (kcal)	1055.05	4057	773.85	4057
Energy	%	%	%	%
Protein	19.2	20	26.2	20
Carbohydrate	67.3	70	26.3	20
Fat	4.3	10	34.9	60
Energy, kcal/g	3.85		5.24	
